# Improved Anaerobic Fermentation of Wheat Straw by Alkaline Pre-Treatment and Addition of Alkali-Tolerant Microorganisms

**DOI:** 10.3390/bioengineering2020066

**Published:** 2015-04-15

**Authors:** Heike Sträuber, Franziska Bühligen, Sabine Kleinsteuber, Marcell Nikolausz, Katharina Porsch

**Affiliations:** UFZ—Helmholtz Centre for Environmental Research, Department of Environmental Microbiology, (in cooperation with) Deutsches Biomasseforschungszentrum (DBFZ), Permoserstr. 15, 04318 Leipzig, Germany; E-Mails: franziska.heine@ufz.de (F.B.); sabine.kleinsteuber@ufz.de (S.K.); marcell.nikolausz@ufz.de (M.N.); katharina.porsch@ufz.de (K.P.)

**Keywords:** lignocellulose, hydrolysis, volatile fatty acids, T-RFLP fingerprinting, Ca(OH)_2_, bioaugmentation, BMP, leach-bed reactor, anaerobic digestion, pre-treatment

## Abstract

The potential of two alkali-tolerant, lignocellulolytic environmental enrichment cultures to improve the anaerobic fermentation of Ca(OH)_2_-pre-treated wheat straw was studied. The biomethane potential of pre-treated straw was 36% higher than that of untreated straw. The bioaugmentation of pre-treated straw with the enrichment cultures did not enhance the methane yield, but accelerated the methane production during the first week. In acidogenic leach-bed fermenters, a 61% higher volatile fatty acid (VFA) production and a 112% higher gas production, mainly CO_2_, were observed when pre-treated instead of untreated straw was used. With one of the two enrichment cultures as the inoculum, instead of the standard inoculum, the VFA production increased by an additional 36% and the gas production by an additional 110%, again mainly CO_2_. Analysis of the microbial communities in the leach-bed processes revealed similar bacterial compositions in the fermenters with pre-treated straw, which developed independently of the used inoculum. It was suggested that the positive metabolic effects with the enrichment cultures observed in both systems were due to initial activities of the alkali-tolerant microorganisms tackling the alkaline conditions better than the standard inocula, whereas the latter dominated in the long term.

## 1. Introduction

Plant biomass can be converted by anaerobic fermentation to chemicals and renewable energy carriers. In these fields of application, the utilization of substrates rich in lignocellulose is of rising interest due to its high abundance. Furthermore, usage of lignocellulosic waste biomass is considered to be sustainable and, thus, socially accepted. Lignocellulosic substrates are suited as a co-substrate for continuous biogas production, because of their high potential methane yield [1,2], as well as for the production of volatile fatty acids (VFA) [3], which is considered to be an economic alternative to biomass saccharification [4]. However, for an effective utilization of these substrates by anaerobic fermentation, many economic and technical obstacles are still to be overcome. A major challenge is the recalcitrance of lignocellulose against microbial decomposition [5]. 

Straw is one of the main lignocellulosic wastes produced during agricultural cultivation of crops. In Germany, 8–13 million tons of cereal straw are available per year for sustainable use beyond agricultural applications [6]. The anaerobic biodegradation of this fiber-rich plant material is limited, as cellulose and hemicellulose are tightly bound to lignin, which is microbially not degradable under anoxic conditions. Wheat straw, for example, contains 35–45% cellulose, 20–30% hemicellulose and 8–15% lignin [7]. Due to this, high retention times may be required for biogas production from straw by anaerobic digestion (AD). The limited accessibility of lignocellulose for enzymatic attack is also problematic in other technologies used for energy gain from straw, e.g., the production of bioethanol. Many substrate pre-treatment techniques are comparably well established in ethanol production technology and can be adapted for improved anaerobic fermentation [5,8]. 

Various physical, physico-chemical, chemical and biological pre-treatment procedures can be used to increase the accessible surface area, to modify the crystalline structure or partially depolymerize cellulose, to solubilize hemicellulose and/or lignin, to modify the lignin structure [5,9] and, thus, to improve the hydrolysis step of anaerobic fermentation. As stable fermentation processes require specific and constant process parameters, the applicability of treatment procedures can be restricted. The most important physical treatment method for plant material is particle size reduction. It results in an enlargement of the specific surface area and a release of intracellular components [10]. Additionally, cellulose chains are shortened, and their crystallinity is reduced [11,12]. One of the most promising chemical methods is the pre-treatment of lignocellulose with alkalis. This usually leads to dissolution of the lignin part of the plant material and a swelling of the cellulose matrix, resulting in an improved accessibility for enzymes [13]. It belongs to the most effective methods, includes the most promising processes for industrial applications and was shown to be more effective on agricultural residues than on wood materials [14]. However, microorganisms performing anaerobic fermentation of such pre-treated substrate are generally not adapted to strong alkaline conditions. For instance, the pH range in which AD runs best depends on the pH optimum of the methanogens, which is between pH 6.8 and 8.5 [15,16,17]. Above this pH range, inhibition of the biological process occurs [18,19,20]. The start of reactors with feeding directly high amounts of alkaline pre-treated substrate or a shift from the untreated to the pre-treated substrate type may be problematic. This could be circumvented by neutralization of the substrate after alkaline pre-treatment. However, the neutralization step would need additional chemicals and is therefore cost-intensive. Another way could be to decelerate the start-up phase or to decrease the organic loading rate in transitional periods waiting for auto-adaptation of the present microbial community. As an alternative, bioaugmentation, *i.e*., the addition of adapted alkali-tolerant microorganisms to the fermentation process, might shorten the start-up phase or adaptation time and thereby reduce costs. 

Bioaugmentation has been widely studied and applied for the bioremediation of soils, sediments and water contaminated with recalcitrant organic compounds, such as petroleum and its derivatives, polycyclic aromatic hydrocarbons, chlorinated compounds, dyes and colored pollutants, pharmaceutically-active compounds and other pollutants (for a review, see Semrany *et al.* [21]). Bioaugmentation has been also applied in AD to overcome obstacles and to reach the optimal performance of bioprocesses, e.g., during AD of cattle manure [22], a mixture of pig slurry and sweet sorghum [23,24], pig manure or dried parts of Jerusalem artichoke [25] and xylan [26]. 

The aim of this study was to determine the potential of alkali-tolerant, lignocellulolytic environmental enrichment cultures to improve anaerobic fermentation processes of alkaline pre-treated wheat straw. The conversion of alkaline pre-treated and untreated wheat straw predominantly to biogas or organic acids was compared in biomethane potential (BMP) assays and leach-bed experiments. The dynamics of the microbial communities in the leach-bed processes were followed by community profiling based on specific marker genes for bacterial and methanogenic communities. Community structures were studied for further interpretation by multivariate statistics and correlations between the abiotic process parameters, and the dynamics of the microbial communities were analyzed.

## 2. Experimental Section

### 2.1. Substrate Pre-Treatment and Analysis

Winter wheat straw harvested at an area in the south-eastern part of Saxony-Anhalt in 2009 and stored dry at outside temperatures was used as the substrate. The straw was first sheared with a straw mill (Strohhexe; Hirlinger, Germany) to an average stalk length of 3 cm and then milled to an average stalk length of 1 cm using a cutting mill (SM 2000; Retsch, Germany). Although the stalk length reduction is already a pre-treatment process, this kind of straw is named untreated straw in the following, as this paper focuses on the effect of alkaline pre-treatment. Next, 200 g_Fresh·Mass_ straw were mixed with 4.6 L distilled water containing 20 g Ca(OH)_2_ in solution. This corresponds to 39.3 g total solids (TS) of straw per liter of 58.7 mM Ca(OH)_2_ solution. The mixture was stored sealed in a polyethylene container for 24 h at 22 °C. For the determination of the BMP (see below), the straw was incubated for seven days in the Ca(OH)_2_ solution, reaching a pH value of 9.7 of the solution. After the incubation, the pre-treated straw was sieved (mesh size 1 mm) without pressure for 10 min, and the particulate fraction was used for the experiments (see below). 

TS and volatile solids (VS) contents of untreated and of pre-treated wheat straw were analyzed as reported earlier [27], and average values were calculated. Crude protein, crude lipid, non-fiber carbohydrate (NFC), cellulose, lignin and hemicellulose content of untreated and of pre-treated wheat straw (24 h pre-treatment time) were determined in duplicates by extended Weende forage analysis according to standard procedures [28,29].

### 2.2. Inocula

For the bioaugmented BMP assays, two mesophilic, alkali-tolerant, lignocellulolytic enrichment cultures, named S37°C and V37°C (enriched at 37 °C from sediments of Lake Szarvas and Lake Velencei in Hungary, respectively; [30]), were used as inocula. The cultures were cultivated as described by Porsch *et al.* [30] with the exception that 400-mL batches with 4 g of untreated wheat straw were set up. These were inoculated with the sixth transfer of S37°C or V37°C [30]. The modified DSMZ (German Collection of Microorganisms and Cell Cultures) medium 1036 was used: 0.5 g·L^−1^ NH_4_Cl, 0.2 g·L^−1^ KH_2_PO_4_, 0.1 g·L^−1^ MgCl_2_ × 6 H_2_O, 0.2 g·L^−1^ KCl, 1 mL trace element solution SL10 (DSMZ medium 320), 1 mL selenite-tungstate solution (DSMZ medium 385), 1.0 g·L^−1^ Na_2_CO_3_, 7.6 g·L^−1^ NaHCO_3_, 2 g·L^−1^ NaCl, 0.2 g·L^−1^ yeast extract, 0.36 g·L^−1^ cysteine-HCl and 0.5 mg·L^−1^ resazurin. The amount of microorganisms added to the Automatic Methane Potential Test System (AMPTS) reactors corresponded to approximately 1% of the microbial cell number of the inoculum slurry (see below). In order to avoid a too high dilution of the inoculum slurry in the BMP set-ups, the microorganisms were concentrated in fresh growth medium. All equipment and solutions used for this procedure were sterile and anoxic. The culture bottles were opened in an anaerobic chamber (98% N_2_, 2% H_2_; Type B; Coy Laboratory Products Inc., Grass Lake, MI, USA), and liquid and straw were separated by sieving. The cell suspension was centrifuged at 10 °C for 10 min at 7000 × *g* (Heraeus Megafuge 16R; Thermo Scientific Inc., Waltham, MA, USA). In the anaerobic chamber, the supernatant was decanted, and the cell pellet was resuspended in fresh medium. The cell number of the cultures before centrifugation and after resuspension and the cell numbers of the inoculum slurry were determined with a Thoma counting cell chamber (Laboroptik GmbH, Friedrichsdorf, Germany) by phase contrast microscopy (Axioplan-2-Imaging, Zeiss, Jena, Germany).

For the leach-bed fermentation experiments, two different inocula were used. A mixture of different acidogenic percolates from previous experiments with wheat straw as the substrate was used as standard inoculum (StI). In these previous experiments, no methane production had been observed. Alternatively, the enrichment culture S37°C was used instead of StI. Four hundred-milliliter batch cultures of S37°C with 2 g of untreated wheat straw were set up with the same growth medium as described above. The cultures were inoculated with the batch culture prepared for the BMP assay. After incubation, the microbial cells were prepared as described above with the exception that the cell pellet was washed with and resuspended in anoxic tap water to remove residues of the medium. Furthermore, the cells of StI were washed in a corresponding manner. To ensure equal amounts of microbial biomass in the inocula StI and S37°C, optical densities (OD) at 700 nm (spectrophotometer Genesys 10 S, Thermo Scientific Inc., Waltham, MA, USA) and protein contents of the inocula were measured and correlated to each other, respectively. For the Lowry protein assay, 0.5 mL 1 N NaOH were added to 0.5 mL cell suspension, incubated 5 min in a boiling water bath for cell disruption and cooled down to room temperature. Samples were prepared in duplicate. For the blank, water was used instead of the cell suspension. Then, the protein content was analyzed as described by Lowry *et al.* [31]. A calibration curve was prepared based on bovine serum albumin (not shown). Since a significant correlation of OD and protein content was achieved (R^2^ = 0.9953), for all experiments, OD analysis was used to adjust the biomass of both of the inocula to each other. 

### 2.3. BMP Assay

The methane yield of untreated and pre-treated straw with and without added alkali-tolerant microorganisms was determined in batch assays at 37 °C using the AMPTS II (Bioprocess Control, Sweden). The system consists of 15 reactors (500 mL reactor volume each) closed by rubber stoppers with integrated stirrers and heated by a water bath. Each reactor was connected to a CO_2_ absorption unit (glass flask filled with 80 mL 3 M NaOH) and the gas volume measuring device, which additionally measured the ambient pressure and temperature. Five different set-ups were prepared in triplicate: two controls with either untreated or pre-treated straw, one set-up with untreated straw and the microbial culture S37°C and two set-ups with pre-treated straw and either the microbial culture S37°C or V37°C. The reactors were filled with 3.6 g untreated or 25.0 g of pre-treated straw (pre-treatment time 7 d; TS = 12.1%_Fresh·Mass_, VS = 10.5%_Fresh·Mass_). In the anaerobic chamber, 30 mL of anoxic sterile growth medium (see above) was added to the controls and 30 mL of one of the concentrated microbial cultures to the other set-ups. The bottles were closed, and the mixtures were pre-incubated in the anaerobic chamber for 20.5 h to allow the added microorganisms to get into close contact with the straw. Afterwards, 366.4 g of a 1:1 mixture of sewage sludge and digestate of a full-scale biogas plant running with maize silage and cow manure were added to each reactor outside the anaerobic chamber. Prior to addition, this inoculum slurry (pH value 7.5, TS = 2.7%_Fresh·Mass_, VS = 1.5%_Fresh·Mass_) was incubated at 37 °C for at least seven days for degassing. Each reactor was connected with the other units of the AMPTS, and the headspace of each reactor and its peripheral installation were flushed with 300 mL N_2_. The reactors were heated up to 37 °C for 30 min before the measurement started. The produced gas volume was measured online and was automatically corrected for the N_2_ present in the system and normalized to standard conditions (p = 101.325 kPa, T = 273.15 K, no humidity).

### 2.4. Reactor Operation

Glass columns (1.65 L total volume) filled with wheat straw were used as leach-bed fermenters with percolation for anaerobic batch fermentation. The installation and operational conditions were as described elsewhere [27] with modifications as outlined in the following. A mesophilic temperature at 38 °C was achieved by operating the reactors in a temperature-controlled chamber (UB 67660; Thermo Electron LED GmbH, Germany). A condensation flask was additionally installed in the gas line between the reactor and the gas volume measurement device (Milligascounter MGC-1 V3.1; Ritter Apparatebau, Germany) outside the chamber. To each column, 50 g_VS_ substrate, *i.e*., untreated or alkaline pre-treated wheat straw, were added. As the basis of the percolation liquid, 1000 mL of anoxic tap water was used. In case of the digestion of pre-treated substrate, the volume of the process liquid was reduced by that amount, which was present in the substrate originating from the pre-treatment process, to ensure equal solid:liquid ratios in all experiments. Thirty milliliters of StI or S37°C (see Section 2.2) were injected. No pH adjustment was carried out. Percolation was operated by sequential pumping of process liquid with an average flow of 200 mL·h^−1^ throughout the whole experimental period. Two reactors per experiment were run in parallel to ensure the reproducibility of the data.

As a reference system (indicated as R), untreated straw was digested using the inoculum StI. The potential of StI to digest pre-treated substrate was evaluated in an experiment indicated as P1, whereas inoculum S37°C was used at two different concentrations for the digestion of pre-treated straw (experiments set up with lower and higher concentrations of microorganisms were indicated as P2 and P3, respectively). The experimental set-up is summarized in Table 1. 

**Table 1:** Leach-bed reactor experiments for the anaerobic fermentation of wheat straw and the used inocula with their biomass concentrations. StI, standard inoculum.

Experiment	Substrate type	Inoculum	OD_700_ of the inoculum
R (Reference)	Untreated	StI	0.163
P1	Pre-treated	StI	0.163
P2	Pre-treated	S37°C	0.160
P3	Pre-treated	S37°C	0.293

### 2.5. Analysis of Process Parameters

The pH values of liquid samples were measured using a pH meter (pH 211; Hanna Instruments, Vöhringen, Germany) equipped with a pH electrode HI 1131B. Concentrations of VFA (acetic, propionic, *n*-butyric, *iso*-butyric, *n*-valeric, *iso*-valeric and *n*-caproic acid) in percolate samples and samples from the AMPTS reactors were determined in triplicate using a 7890 A gas chromatograph (Agilent Technologies) equipped with a TurboMatrix 110 automatic headspace sampler (Perkin Elmer), an HP-FFAP column (0.25 µm × 0.32 mm × 30 m, Agilent Technologies) and a flame ionization detector. Nitrogen was the carrier gas with a flow rate of 8.5 mL·min^−1^. The chromatographic conditions were as follows: injector temperature, 220 °C (split-splitless); detector temperature, 260 °C; and an oven temperature program initiating at 40 °C, followed by three sequenced temperature increases (i) at a rate of 60 K·min^−1^ up to 100 °C, (ii) at a rate of 50 K·min^−1^ up to 150 °C and, finally, (iii) at a rate of 90 K·min^-1^ until 240 °C was reached. Five milliliters of supernatant of a centrifuged sample were filled into a 20-mL glass vial. If the concentration of an organic acid was higher than 10 g·L^−1^, the sample was diluted 1:5 in distilled water prior to measurement. To each vial, 1 mL of 42.5% phosphoric acid and 1 mL internal standard (2-ethylbutyric acid; 184 mg·L^−1^ stock solution) were added. The vials were incubated for 32 min at 85 °C before injection. Average values were calculated from the triplicates. Volumes of produced gas were measured on-line using Milligascounters MGC-1 V3.1. The composition of the gas was analyzed in duplicate by gas chromatography using a CP-2002 P micro gas chromatograph (GC; Chrompack) equipped with thermal conductivity detectors and two fixed columns (Column A, Molsieve 5 Å PLOT for separating H_2_, N_2_, and CH_4_; and Column B, HayeSep A for separating CO_2_) running in parallel, and average values were calculated. The carrier gas was argon. The chromatographic conditions were as follows: the temperature of Column A was set to 30 °C, and the appropriate detector mode was set to low sensitivity, whereas the temperature of Column B was 50 °C, and the running mode of the detector was set to high sensitivity. A 1-mL gas sample was injected into a 20-mL GC vial filled with argon. The injection time in the GC was set to 5 ms. The four gases were detected in significant amounts depending on the reactor. Since nitrogen was used as the cover gas in the reactors to ensure anoxic conditions and was not produced during the process, the detected concentrations of the other gases were summarized and set to 100%.

### 2.6. Microbial Community Analysis

The composition of the microbial community was analyzed by terminal restriction fragment length polymorphism (T-RFLP) fingerprinting of bacterial 16S rRNA genes or *mcrA* genes (encoding methyl coenzyme M reductase as a specific marker for the methanogenic archaea). Percolate samples were centrifuged for 5 min at 20,817 × *g* (Centrifuge 5417R; Eppendorf, Germany). The pellets were washed in 100 mM phosphate-buffered saline pH 7.5 and frozen at −20 °C until DNA extraction.

Total DNA was extracted using the NucleoSpin^®^ Tissue Kit (Machery-Nagel, Düren, Germany) according to the manufacturer’s protocol for hard to lyse bacteria, with the following exception: instead of 20 mg·mL^−1^ lysozyme, only 3 mg·mL^−1^ were used. DNA concentration and quality were photometrically determined using a NanoDrop^®^ ND-1000 UV-Vis spectral photometer (Thermo Fisher Scientific Inc., MA, USA) and by agarose gel electrophoresis. 

For community analysis, T-RFLPs were analyzed as described previously [27] with some modifications. Bacterial 16S rRNA gene fragment polymerase chain reaction (PCR) was carried out in 25.0 μL reaction mixtures containing 10 ng template DNA, 5 pmol of each primer and 12.5 μL of Taq Master Mix (QIAGEN, Hilden, Germany). The cycle parameters were as follows: an initial denaturation at 94 °C for 4 min, 35 cycles of 45 s at 94 °C, 1 min at 58 °C, 2 min at 72 °C and a final elongation at 72 °C for 20 min. Purified PCR products were quantified using a NanoDrop^®^ ND-1000 UV-Vis spectral photometer (Thermo Fisher Scientific Inc., MA, USA) and digested with the restriction endonucleases *Hae*III and *Msp*I (New England Biolabs GmbH, Frankfurt/Main, Germany), respectively, using 2 U of the respective enzyme for digesting 20 ng PCR product. The samples were incubated at 37 °C overnight. After ethanol precipitation, dried DNA samples were resuspended in 10 μL HiDi formamide (Applied Biosystems GmbH, Weiterstadt, Germany) containing 1.5% (v/v) MapMarker^®^ 1000 (BioVentures Inc., Murfreesboro, TN, USA) labelled with 5-carboxy-X-rhodamine (ROX).

PCR amplification of *mcrA* gene fragments was done according to Steinberg and Regan [32] using the forward primer mlas (5ʹ-GGT GGT GTM GGD TTC ACM CAR TA-3ʹ; Eurofins MWG Operon, Ebersberg, Germany) and the reverse primer mcrA-rev_FAM (5ʹ-CGT TCA TBG CGT AGT TVG GRT AGT-3ʹ; Eurofins MWG Operon); the latter was labelled with 6‑carboxyfluorescein (FAM). PCR was carried out in 12.5-μL reaction mixtures containing 10 ng template DNA, 5 pmol of each primer and 6.25 μL of My Taq HS Red 2× Mix (Bioline GmbH, Luckenwalde, Germany). The cycle parameters were as follows: an initial denaturation at 95 °C for 3 min, 5 cycles of 30 s at 95 °C, 45 s at 48 °C, 30 s with a ramp rate of 0.1 K·s^−1^ to 72 °C, followed by 25 cycles of 20 s at 95 °C, 20 s at 55 °C, 15 s at 72 °C and a final elongation at 72 °C for 10 min. Purified PCR products were quantified using a NanoDrop^®^ ND-1000 UV-Vis spectral photometer (Thermo Fisher Scientific Inc., Waltham, MA, USA) and digested with the restriction endonuclease *Bst*NI (New England Biolabs GmbH, Frankfurt/Main, Germany), using 1 U of the enzyme for digesting 10 ng of the PCR product. The samples were incubated at 60 °C for 2 h. Precipitation and resuspension of dried DNA samples were done as described above for 16S rRNA gene analysis, but as the size standard, the GeneScan^TM^-500 ROX^TM^ (Applied Biosystems GmbH, Weiterstadt, Germany) was used.

### 2.7. Statistical Analysis

Multivariate statistical analysis using the normalized sample-peak table was performed with the software R Version 3.0.1 using the R package “vegan” [33] as previously described [27]. Non-metric multidimensional scaling (NMDS) analyses applying the Bray–Curtis dissimilarity index (regarding the presence and relative abundance of peaks) were used to plot the rank order of dissimilarity of T-RFLP profiles in a way that distances are exactly expressed on a two-dimensional sheet (greater distances represent greater dissimilarities). T-RFLP results of both of the parallel assays, respectively, were included in this analysis. The major process parameters correlating with community composition, as well as with single terminal restriction fragments (T-RF) were fitted using the “envfit” algorithm provided with the “vegan” package. Significance (*p* < 0.001) of single process parameters on the NMDS results were tested using a Monte-Carlo test with 999 permutations. For statistical analysis, T-RFLP datasets were reduced by removing T-RF with abundances below 1% in every sample and with less than four detections in all samples. 

## 3. Results 

### 3.1. Effect of Alkaline Pre-Treatment on Substrate Characteristics

Wheat straw is a feedstock rich in lignocellulose, as reflected by the predominant fraction of fiber (cellulose, lignin and hemicellulose) in the extended Weende forage analysis (Table 2). As alkaline pre-treatment is a wet pre-treatment technique, the TS content of pre-treated straw was much lower than that of untreated straw. The slightly lower VS content of pre-treated straw is probably caused by the increased amount of inorganics, *i.e*., residues of chemicals from the pre-treatment procedure. The lignin and cellulose contents were not considerably changed by the pre-treatment process, whereas the hemicellulose content decreased by 18.2%. The contents of fractions with a minor proportion were decreased (crude protein and crude lipid) or increased (NFC) by the pre-treatment process.

**Table 2:** Extended Weende forage analysis of wheat straw (pre-treatment time: 24 h). In the case of duplicates, the differences of average values from minimum and maximum are indicated in brackets. In the case of triplicates, the range of values is given. TS, total solids; VS, volatile solids; NFC, non-fiber carbohydrates.

Parameter	Untreated wheat straw	Alkaline pre-treated wheat straw
TS (%_Fresh·Mass_)	90.4 ^*^	14.0 (13.2–14.8)
VS (%_TS_)	91.1 ^*^	85.2 (85.0–85.4)
Ash (g·kg_TS_^−1^)	88.6 ^*^	148.4 (146.0–150.2)
Crude protein (g·kg_TS_^−1^)	63.0 (±0.5)	54.7 (±0.3)
Crude lipid (g·kg_TS_^−1^)	16.8 (±0.4)	7.2 (±0.2)
NFC (g·kg_TS_^−1^)	33.1 (±2.6)	38.6 (±1.7)
Cellulose (g·kg_TS_^−1^)	466.1 (±0.2)	460.5 (±2.6)
Hemicellulose (g·kg_TS_^−1^)	237.7 (±4.7)	194.4 (±4.5)
Lignin (g·kg_TS_^−1^)	94.7 (±3.2)	96.3 (±0.6)

^*^ Value from a single measurement. The errors of TS, VS and ash measurements of other samples from the same straw batch in duplicates or triplicates were maximally 0.6%.

### 3.2. Effect of Alkaline Pre-Treatment and Bioaugmentation on the Biomethane Potential

**Figure 1:**
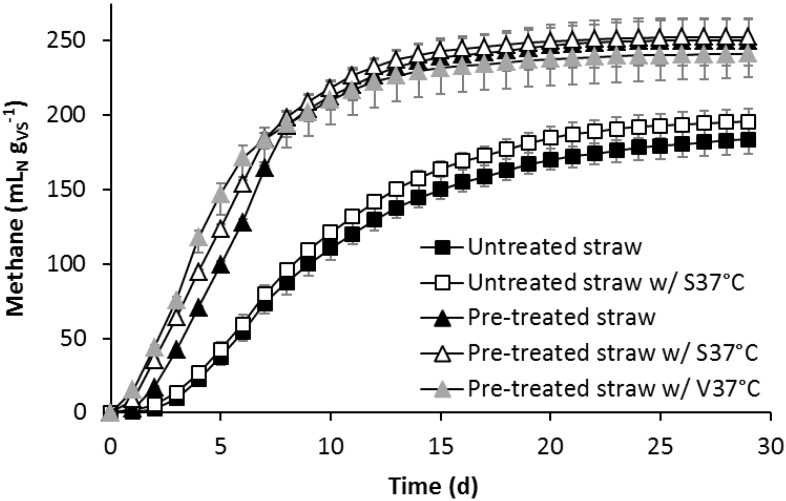
Methane production from untreated and alkaline pre-treated straw with and without the addition of the alkali-tolerant, lignocellulolytic enrichment culture S37°C or V37°C, respectively, determined in batch assays. The methane volume refers to the VS content of the straw. Values are averages of triplicates, except “untreated straw w/S37°C”, for which the averages of duplicates are shown. Bars indicate the differences from the average to the minimum and maximum value.

**Figure A1:**
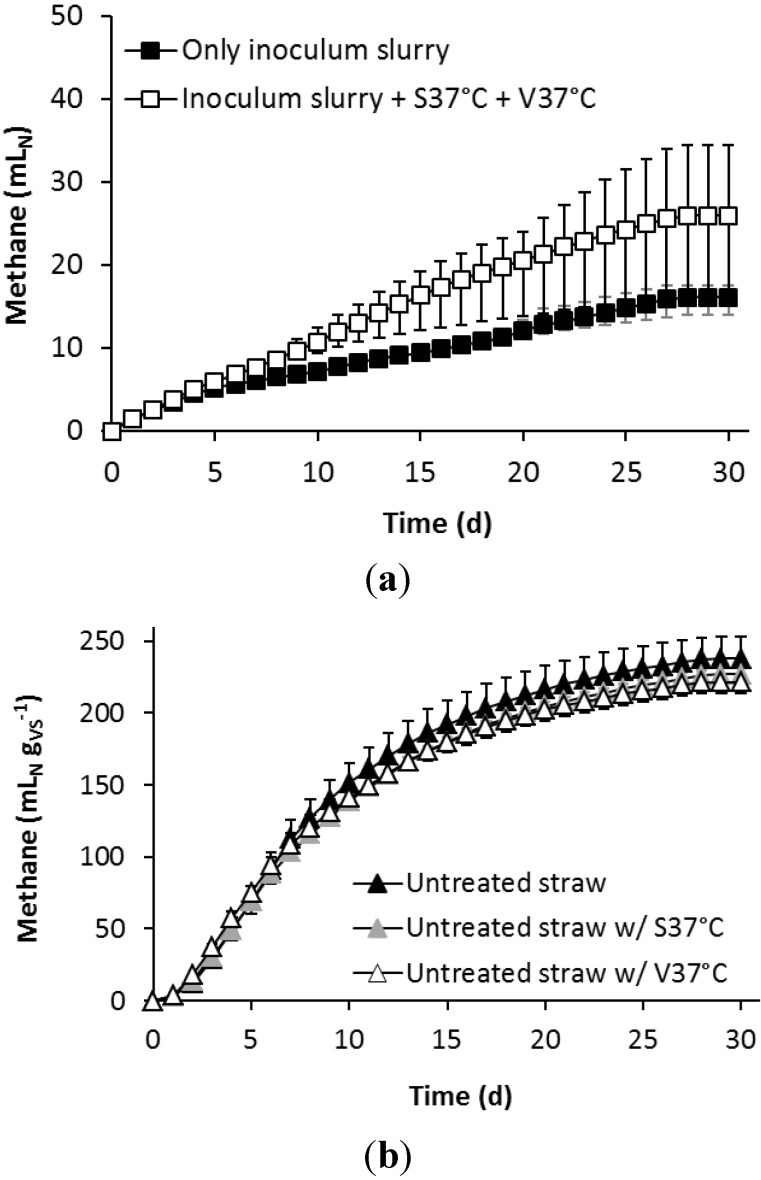
(**a**) Methane production from the inoculum slurry alone and from the inoculum slurry plus a mixture of the cultures S37°C and V37°C determined in batch assays. Since no substrate was added, only the produced methane volume is depicted. Values are averages of triplicates. Bars indicate the difference from the average to the minimum and maximum value measured. The methane production from the inoculum slurry varied between 14 and 18 mL_N_; with the addition of S37°C and V37°C, it was between 17 and 34 mL_N_. Based on the average values, the difference of the methane volumes between the two set-ups was 10 mL_N_ after 30 days. This corresponds approximately to the smallest gas volume that the AMPTS can detect. (**b**) Methane production from untreated straw and from untreated straw with the addition of the alkali-tolerant, lignocellulolytic enrichment culture S37°C or V37°C, respectively, determined in batch assays. The methane volume refers to the VS content of the added straw. Values are averages of triplicates. Bars indicate the difference from the average to the minimum and maximum value measured.

The methane yield of untreated or pre-treated straw with or without added alkali-tolerant, lignocellulolytic enrichment cultures S37°C or V37°C was determined in batch assays. The results refer to the VS content of straw. The methane production from untreated straw was slower and much lower than that from pre-treated straw (Figure 1). After 29 days of incubation, 183 mL_N_ methane g_VS_^−1^ was formed as the average in set-ups with untreated straw, whereas set-ups with pre-treated straw yielded 250 mL_N_ methane g_VS_^−1^, representing an increase of 36%. The addition of culture S37°C to untreated straw led to a slightly faster methane production right from the start. From Day 10 on, the methane yield of the set-up with S37°C was 11–15 mL_N_·g_VS_^−1^ higher than that of the set-up with untreated straw alone. In a second BMP assay, similar results were obtained, where the addition of S37°C or V37°C had no effect on methane production from untreated straw (Appendix, Figure A1b). 

In contrast, the addition of cultures S37°C and V37°C to pre-treated straw led to a much higher methane production within the first week, but after one week, the methane production was the same (S37°C) or even lower (V37°C) than in the set-up with pre-treated straw alone (Figure 1). The maximum difference between pre-treated straw without bioaugmentation and pre-treated straw with S37°C microorganisms was reached on Day 6 with 26 mL_N_ methane g_VS_^−1^, corresponding to an increase of 20%. From Day 8 on, the bioaugmented set-up produced only 2–7 mL_N_·g_VS_^−1^ more methane. The maximum increase in methane production by the addition of the V37°C culture to pre-treated straw occurred on Day 5 with 47 mL_N_·g_VS_^−1^ more methane, representing an increase in methane production of 48%. However, during the last two weeks, 8–10 mL_N_·g_VS_^−1^ less methane was formed compared to pre-treated straw without bioaugmentation.

In the second BMP assay, the inoculum slurry alone produced 16 mL_N_ methane within 30 days. The addition of a mixture of S37°C and V37°C to the slurry resulted only in 10 mL_N_ more methane. The maximum difference between the bioaugmented and non-bioaugmented set-ups with pre-treated straw during the first week corresponded to 68 mL_N_ (S37°C) and 125 mL_N_ (V37°C) methane.

On the first day, the pH value was measured in one reactor of the AMPTS with untreated and in one reactor with pre-treated straw, and the pH values in both reactors were 7.7. After 29 days of incubation, the pH values of all reactors were similar and varied between 7.4 and 7.5. Furthermore, the organic acid concentrations were similar in all reactors at the end of the experiment. The concentrations of total organic acids were 9–13 mg·L^−1^, which is very low. The main organic acid was acetic acid at 7–10 mg·L^−1^. Traces of *n*-caproic acid (<2 mg·L^−1^) were detected in all reactors, whereas propionic acid (<3 mg·L^−1^) was only detected in half of the reactors and *iso*-butyric acid (<1 mg·L^−1^) only in one reactor. Neither *n*-butyric nor *iso*- or *n*-valeric acid were detected in any of the set-ups.

### 3.3. Leach-Bed Fermentation

Untreated or pre-treated straw was used for anaerobic batch fermentation experiments using the standard inoculum (StI) or the alkali-tolerant, lignocellulolytic enrichment culture S37°C for inoculation. The culture S37°C was chosen over V37°C, because of its better performance during the BMP assay. 

**Figure 2:**
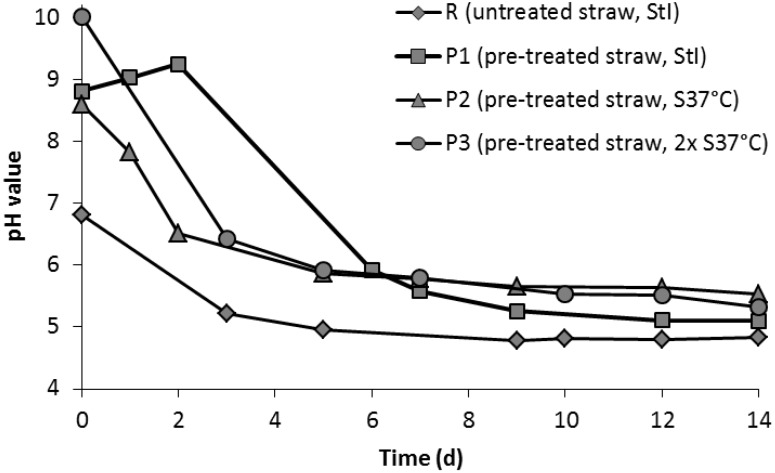
pH values of the percolate during the fermentation of untreated (R) and pre-treated (P1, P2 and P3) straw with the inocula StI or S37°C (see Table 1).

The starting pH values of the four experiments (see Table 1) varied depending on the type of substrate used. With untreated straw, the starting pH of the percolate was nearly neutral at 6.8. In contrast, when pre-treated straw was used, the starting pH was in the alkaline range above eight (Figure 2). In experiment P1, the pH value of the percolate increased even further within the first two days, since Ca(OH)_2_ was washed out from the substrate, indicating a lower metabolic activity of inoculum StI at the beginning. Independently of the starting pH, the pH value dropped in all experiments to 5.5 or below during fermentation. The pH value of the reference experiment was 4.8, the lowest pH value of all assays at the end of the experiment. 

**Figure 3:**
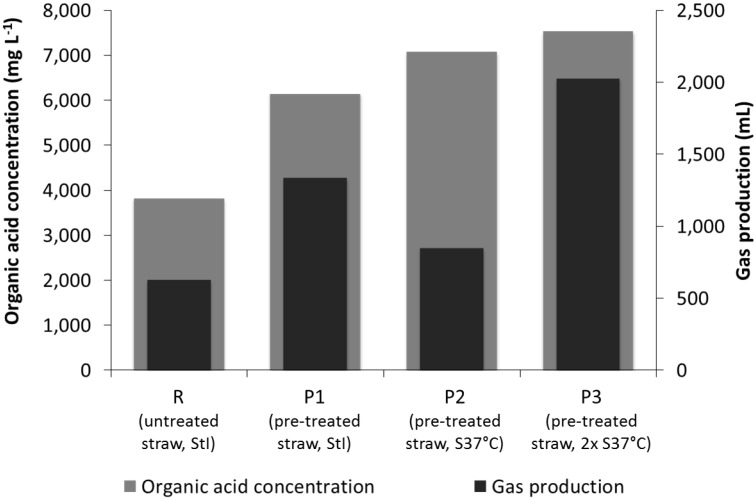
Average gas production and organic acids (sum of volatile fatty acids) concentration after 14 days of fermentation of untreated (R) and pre-treated (P1, P2 and P3) straw with the inocula StI or S37°C (see Table 1).

In all experiments, straw was fermented to typical primary liquid and gaseous fermentation products indicating that hydrolytic and acidogenic processes dominated. Figure 3 summarizes the organic acid and gas production values measured in the single experiments. The alkaline pre-treatment of the straw resulted in a higher yield of organic acids and increased gas formation during its digestion compared to the digestion of untreated straw. The gas production differed between all four experiments. The least gas was produced in the reference experiment R (in total 630 mL), whereas 112% more gas (in total 1337 mL) was produced with pre-treated straw (P1). In the experiments where S37°C was used as the inoculum, the gas production was 35% higher (in total 848 mL; P2) or, with double the concentration of microorganisms in the inoculum, even 222% higher (2030 mL; P3) compared to the reference. 

**Figure 4:**
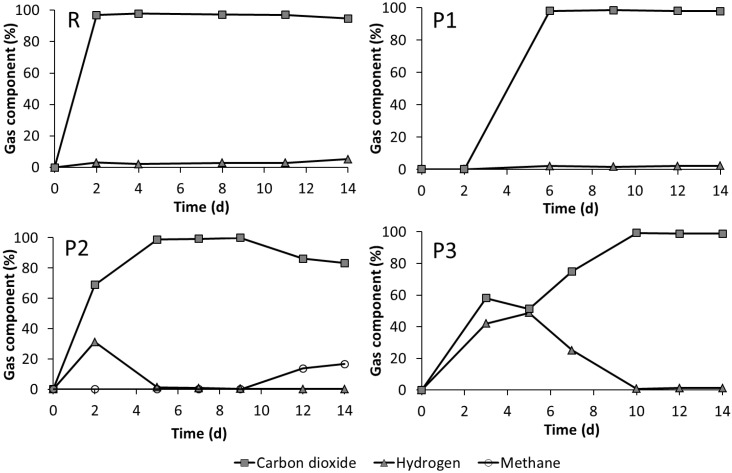
Composition of the gasses produced during fermentation of untreated straw with StI (R), pre-treated straw with StI (P1), pre-treated straw with S37°C (P2) and pre-treated straw with double the amount of S37°C (P3) (see Table 1).

**Figure A2:**
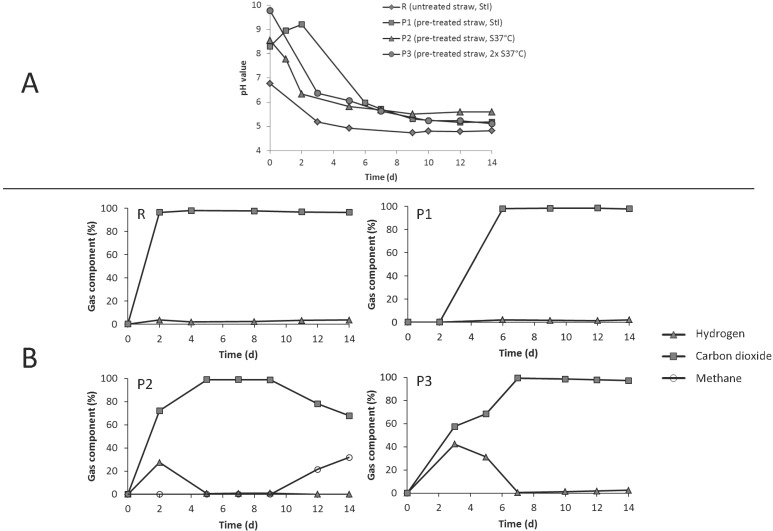
pH values (**A**) and composition of the gasses produced (**B**) during anaerobic fermentation of untreated (R) and pre-treated (P1, P2 and P3) straw with the inocula StI or S37°C in the parallel experiments.

In most experiments, the produced gas was composed solely of hydrogen and carbon dioxide (Figure 4). With StI (experiments R and P1), the hydrogen concentration did not exceed 5%. In contrast, with S37°C as the inoculum, the hydrogen concentration was much higher, with maximum concentrations of 31% on Day 2 in P2 and 48% on Day 5 in P3, indicating differences in the fermentation performance of S37°C compared to the microorganisms of StI. However, in these experiments, the hydrogen concentration dropped after a few days of fermentation to a concentration of 1% or less. The process parameters obtained from the parallel running experiments (included in Appendix, Figure A2) confirmed these results. Methane production was detected only in P2 from the twelfth day on, although the pH value was already in the acidic range after the second day of running (Figure 2 and Figure 4). At the end of fermentation, the gas produced in the P2 duplicate experiments contained 17% (Figure 4) and 32% (Figure A2) methane, respectively. 

**Figure 5:**
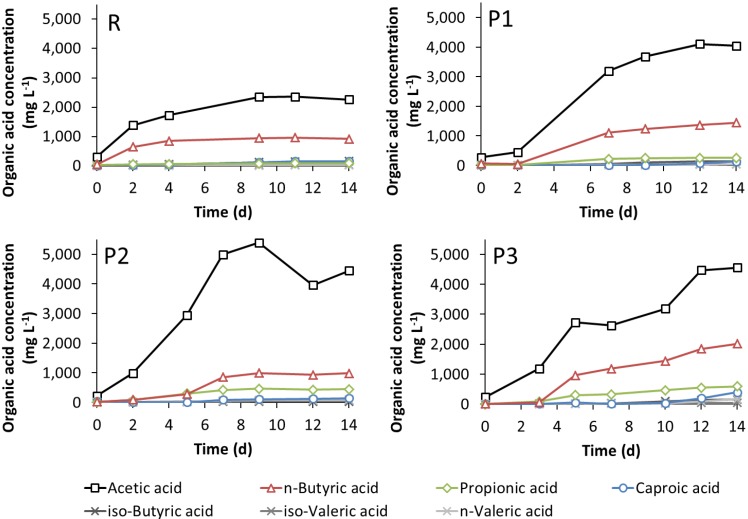
Production of VFA during the fermentation of untreated straw with StI (R), pre-treated straw with StI (P1), pre-treated straw with S37°C (P2) and pre-treated straw with double the amount of S37°C (P3) (see Table 1).

**Figure A3:**
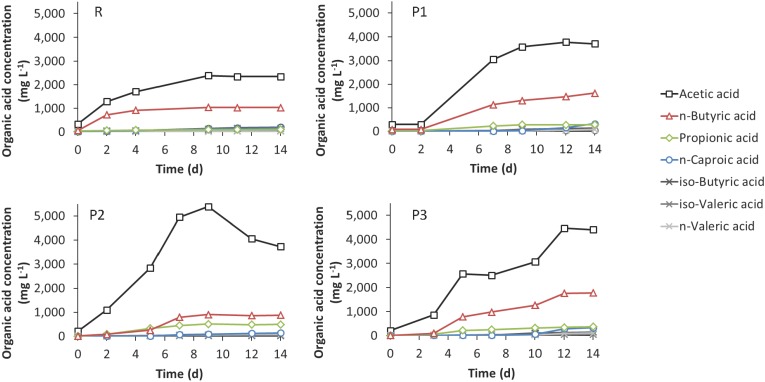
Production of VFA during the fermentation of untreated straw with StI (R), pre-treated straw with StI (P1), pre-treated straw with S37°C (P2) and pre-treated straw with double the amount of S37°C (P3) in the parallel experiments.

Additionally, the organic acid production varied among the four experiments. Mainly acetic acid and *n*-butyric acid were produced when StI was used as the inoculum (Figure 5; experiments R and P1). Furthermore, propionic, *iso*-butyric, *n*- and *iso*-valeric, as well as *n*-caproic acid were detected in low concentrations below 160 mg·L^−1^ in experiment R. When pre-treated straw was used as the substrate (P1), 64% more acetic acid (up to 4110 mg·L^−1^) and 76% more *n*-butyric acid (up to 1440 mg·L^−1^) were produced from 50 g_VS_ straw compared to the digestion of untreated straw (R), respectively. With S37°C as the inoculum (experiments P2 and P3), the production of organic acids was even higher (maximum 5390 mg·L^−1^ acetic acid in P2 and 2010 mg·L^−1^ *n*-butyric acid in P3) compared to the experiments R and P1. The production of propionic acid was likewise increased (up to 590 mg·L^−1^) in P2 and P3, as well as the production of *n*-caproic acid (up to 400 mg·L^−1^) at the end of fermentation in P3. In contrast, the concentrations of *iso*-butyric and *iso*-, as well as *n*-valeric acid did not considerably change (maximum 180 mg·L^−1^). The acetic acid concentration in P2 decreased after nine days of fermentation, probably because of methane production (Figure 4), as both events were simultaneously observed. Interestingly, the acetic acid concentration in P3 did not increase compared to P2, as was detected with *n*-butyric acid. The VFA production in the parallel running experiments confirmed the results (see Appendix, Figure A3). 

Altogether, in the experiment P1, with pre-treated straw, the microbial community of the standard inoculum StI, although not adapted to the alkaline conditions, showed a surprisingly good performance. Only a lag phase of two days was observed as a sign of adjustment (Figure 2, Figure 4 and Figure 5). The addition of a microbial community adapted to the alkaline conditions (S37°C) resulted in even higher yields of fermentation products, except of the gas yield in the P2 set-up. 

### 3.4. Dynamics of the Microbial Communities and Correlation with Abiotic Data

**Figure 6:**
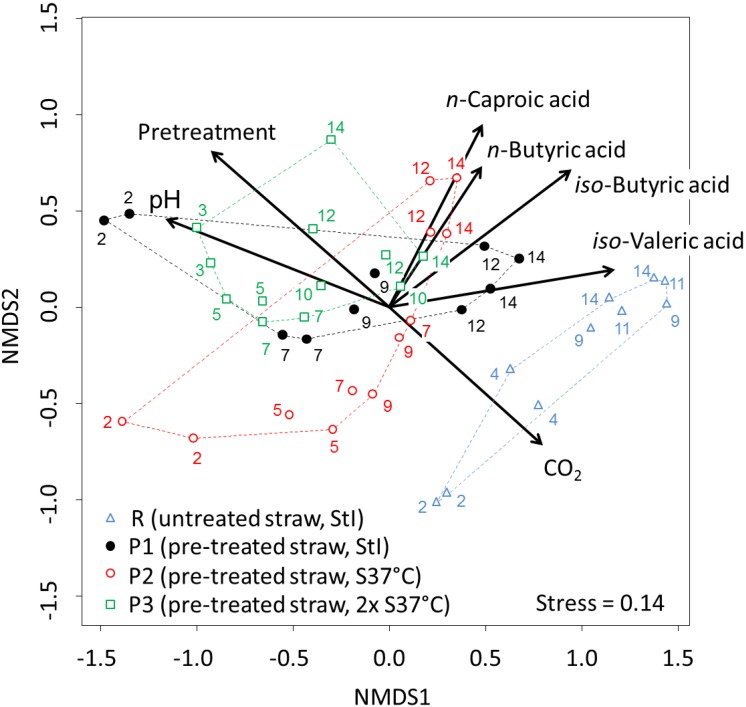
Non-metric multidimensional scaling (NMDS) analysis plot of T-RFLP profiles of bacterial 16S rRNA amplicons digested with the restriction enzyme *Hae*III. Community dissimilarity is based on the Bray–Curtis index, which includes the presence and relative abundance of T-RF. Sampling times of the duplicate experiments are indicated next to the symbols. T-RFLP profiles of the experiments R, P1, P2 and P3 (samples from duplicate experiments) are indicated by different symbols of different colors and enclosed by hulls, respectively. Arrows indicate the correlation vectors of community differences and the process parameters with significance factors *p* < 0.001.

**Figure A4:**
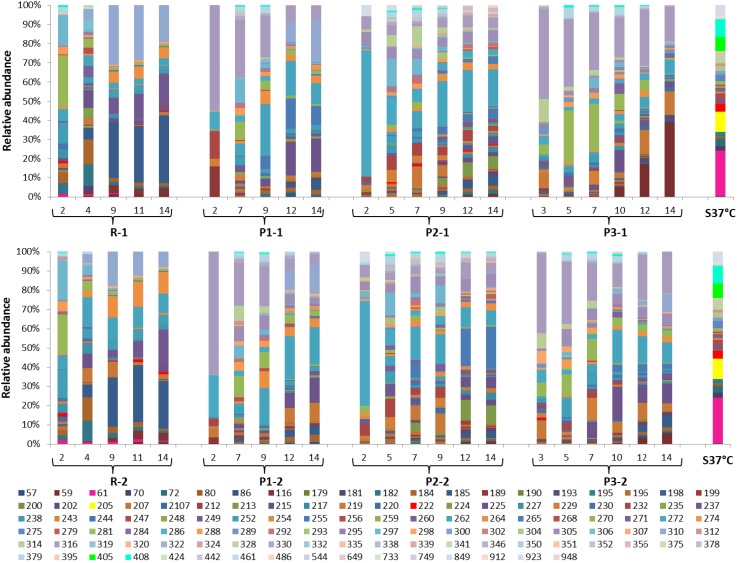
T-RFLP patterns of the bacterial communities of the experiments (duplicate experiments indicated as -1 and -2) and enrichment culture S37°C obtained with the restriction enzyme *Hae*III. In the community S37°C, 36 T-RF were detected, but 13 of those were not found in P2 or P3. Ten T-RF from S37°C were present in P2, but not in P3. Another 13 T-RF were present in S37°C, as well as in P2 and P3. The three most abundant T-RFs, 61, 205 and 408, in S37°C were not detected in P2 and P3 or only with minor abundances. Additionally, the most abundant T-RFs of P2 and P3, 59, 262, 270, 272 and 316, were not detected or only to a minor abundance present in S37°C.

**Figure A5:**
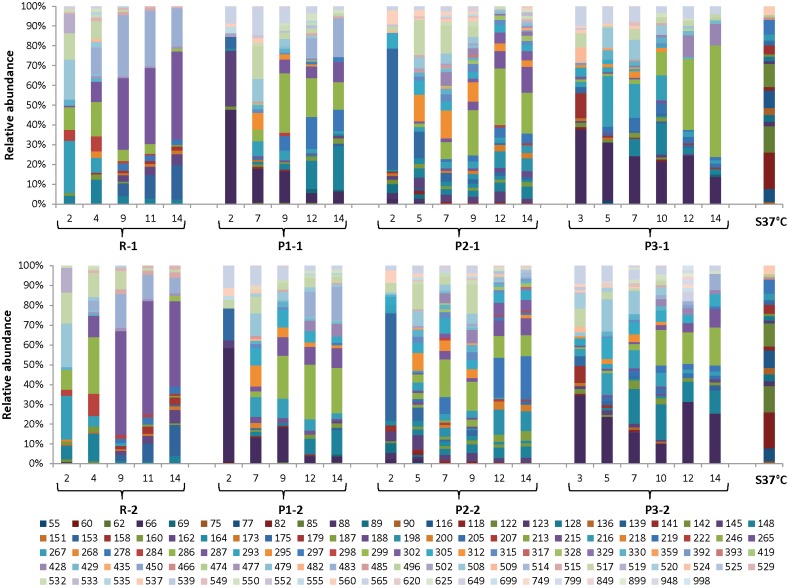
T-RFLP profiles of bacterial 16S rRNA amplicons in the leach-bed experiments (running in duplicates indicated as -1 and -2) and the enrichment culture S37°C. *Msp*I was used as the restriction enzyme.

In parallel to the process analytics, the compositions of bacterial (all leach-bed experiments) and methanogenic (only P2 experiment) communities were monitored over time by T-RFLP fingerprinting of cells harvested from the process liquids. The biotic and abiotic data were statistically analyzed to identify relationships between the different parameters. The dynamics of the microbial communities based on their T-RFLP profiles and their correlations with process parameters are visualized in an NMDS plot shown in Figure 6. The NMDS plot indicates similarity or dissimilarity of the bacterial communities of the experiments R–P3 to each other. Interestingly, the bacterial communities digesting pre-treated straw (P1, P2 and P3) clustered together independently of the used inoculum. In contrast, the bacterial community of the reference experiment (R), where untreated straw was used as the substrate, was relatively dissimilar to the bacterial communities of the experiments with pre-treated straw. Furthermore, the composition of the bacterial communities in the respective experiments changed during cultivation. This was eminent in the case of R, P1 and P2, where strong community shifts were detected. In the case of the P3 experiment, the shift was less pronounced. In this experiment, the first sample for analysis of the bacterial community was taken three days after starting the experiment and not after two days, as was done in the other experiments. As the shift of the bacterial communities was biggest at the beginning of the experiments, this could be the reason for the less pronounced shift in P3. The bacterial community composition in the R set-up was correlated with the carbon dioxide content of the hydrolysis gas, as indicated by the CO_2_ vector in the NMDS plot. The vector for the pH value points to the opposite direction, indicating a contrary relationship of these communities and the pH value. Bacterial communities of the late phases of P1, P2 and P3 were significantly correlated with the formation of *n*-butyric, *iso*-butyric, *iso*-valeric and *n*-caproic acid. T-RFLP patterns of the bacterial communities of all four experiments produced with the restriction enzymes *Hae*III and *Msp*I are shown in the Appendix, Figure A4 and Figure A5, respectively. A detailed comparison of the bacterial community composition of the leach-bed experiments P2 and P3 and the enrichment culture S37°C is presented in the Appendix, Figure A4. 

**Figure A6:**
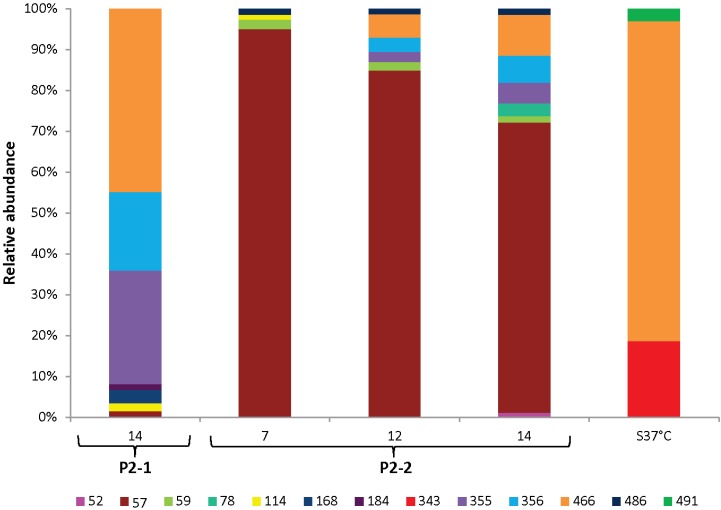
T-RFLP profile of *mcrA* amplicons in the leach-bed experiment P2 (running in duplicate indicated as P2-1 and P2-2), as well as the enrichment culture S37°C. *Bst*NI was used as the restriction enzyme.

Analysis of the methanogenic community in the experiment P2 and of the enrichment culture S37°C revealed changes in the community compositions during fermentation (Appendix, Figure A6). In general, the reactor samples showed more diverse methanogenic communities than the inoculum. In the culture S37°C, three major T-RFs were detected. Only one of them (T-RF 466) was also detected in the reactor samples, whereas the other two (T-RF 343 and 491) were not. The methanogenic communities of the P2 duplicate set-ups differed from each other. Detection of *mcrA* genes from Experiment 1 was successful only on Day 14, whereas on the Days 7 and 12, the DNA concentration of the PCR products was too low for T-RFLP analysis of the methanogens. In Experiment 1, T-RF 466, 355 and 356 were predominant. In Experiment 2, T-RF 57 predominated, while T-RF 466, 355 and 356 showed lower abundance. 

## 4. Discussion

Anaerobic fermentation processes producing biogas as a renewable energy carrier or VFA as platform chemicals are based on equivalent metabolic processes, *i.e.*, hydrolysis of the substrate and acidogenesis from the disintegrated molecules. Alkaline pre-treatment of lignocellulose and subsequent bioaugmentation with alkali-tolerant microorganisms aims at an improved efficiency of these two steps. Due to difficulties in the direct evaluation of hydrolytic performances in systems of high microbiological complexity, the production of end products of the respective processes, *i.e.*, biogas in case of the BMP assay or VFA during anaerobic fermentation in leach-bed reactors, was monitored. 

### 4.1. Effect of Alkaline Pre-Treatment on the AD of Wheat Straw

The chemical composition of the untreated wheat straw used in this study was in good agreement with data on the composition of wheat straw in other studies [34,35,36,37,38]. In many studies, substrate pre-treatment with NaOH is described [14,34,38]. In this study, Ca(OH)_2_ instead of NaOH was used because of its lower price per kg hydroxide. Furthermore, Ca(OH)_2_ could potentially be recovered from the treatment liquid by neutralization with the process gas CO_2_ followed by lime kiln technology [39]. Alkaline pre-treatment of lignocellulosic material aims to remove part of the lignin and hemicellulose by solubilization and to enhance the accessibility of the cellulosic part for cellulolytic enzymes [5,14]. In agreement with this and other literature values [34,36], the pre-treatment of wheat straw led in our study to a substantial decrease of the hemicellulose content, while the cellulose content was only marginally affected. In contrast, the lignin content was not changed by the pre-treatment process. This was probably due to calcium bonding to lignin, which leads to a high remaining lignin content after pre-treatment, but has no influence on the biomass digestibility [40].

The methane yield of untreated wheat straw determined in this study (183 mL_N_·g_VS_^−1^) was at the lower end of methane yield values reported by other researchers (204 mL·g_VS_^−1^ [38], 276 mL_N_·g_VS_^−1^ [35], 297 mL·g_VS_^−1^ [37]). In some other studies, even a methane yield below 100 mL g_VS_^‑1^ was measured for untreated wheat straw [34,36], at which Pavlostathis and Gossett [34] stated that the digestion of the straw was not completed. The reasons for the differences between the studies could be that different test systems, inoculum slurries of different origins, different substrate to inoculum ratios and different incubation temperatures were used. Furthermore, our straw particles had a length of 10 mm, whereas in the other studies [34,35,36,37,38], straw particles of 1–3 mm in size were used. The reduction in particle size is an additional mechanical pre-treatment method leading to a better hydrolysis of the straw and a higher methane yield [5,14], which might explain the higher methane yield determined by other researchers. 

The improvement of the methane yield (36%) by alkaline pre-treatment in our study was in a similar range as in the study of Sambusiti *et al.* [38], who used a similar set-up, but applied NaOH for the pre-treatment. In other studies, the reported methane yield improvement was much higher [34,36]. Since different factors affect the alkaline pre-treatment and the BMP assay, it is difficult to identify one key factor being responsible for the discrepancy between the different studies. Furthermore, it was not our intention to develop an optimized alkaline pre-treatment, but to study the effect of bioaugmentation on the hydrolysis of alkaline pre-treated wheat straw.

### 4.2. Effect of Bioaugmentation on the AD of Alkaline Pre-Treated Wheat Straw

The addition of alkali-tolerant microorganisms to untreated straw led only to a very small or even no increase in the methane yield of the two BMP assays. This was not surprising, since the mixture of straw and inoculum slurry had no elevated pH value, and hence, the alkali-tolerant microorganisms did not have any advantage over the microorganisms present in the inoculum slurry. 

In the case of the alkaline pre-treated straw, the final methane yields of the set-ups with and without bioaugmentation were also similar. However, during the first week of the experiment, the methane was produced faster in the bioaugmented set-ups. The difference of final methane volumes with and without a mixture of S37°C and V37°C (without straw) was low compared to the difference in the methane volumes produced by bioaugmented and non-bioaugmented set-ups with pre-treated straw in the first week. Therefore, it can be assumed that the positive effect of the bioaugmentation was not due to the digestion of the added microorganisms, but rather to their activity. All in all, the relationship of the cost to the benefit of the bioaugmentation procedure is questionable. In contrast to other studies [34,38], no pH adjustment of the alkaline pre-treated straw (pH 9.7) was performed in the present study before mixing it with the inoculum slurry. However, due to the alkalinity of the slurry, no increase in its pH was observed after mixing it with the pre-treated substrate, indicating that the microorganisms present in the inoculum slurry were probably not affected by the high pH of the pre-treated straw. However, when a fast process with low retention times is applied, bioaugmentation might be helpful. Additionally, when alkaline pre-treated straw is used as the substrate in continuously running systems, the daily addition of alkali might exceed the buffering capacity of the system, leading to increased pH values. In such a case, the addition of alkali-tolerant microorganisms might be an alternative to a slow start-up phase or low organic loading rates in transitional periods aiming at the auto-adaptation of the present microbial community. Nevertheless, the positive effect of adding alkali-tolerant, lignocellulolytic microorganisms to continuous systems still needs to be proven. 

Bagi *et al.* [25] and Weiß *et al.* [26] also determined the effect of bioaugmentation on the methane and biogas yield in batch assays under mesophilic conditions. In contrast to our approach (enrichment of microorganisms from the environment), Weiß *et al.* [26] first enriched microorganisms from digestate from a biogas plant on xylan, a major constituent of hemicellulose, and later used these microorganisms immobilized on zeolite for a bioaugmentation assay for which the same digestate amended with xylan was used. Bagi *et al.* [25] followed another approach and aimed to increase the biogas yield from sewage sludge, pig manure and Jerusalem artichoke by the addition of a pure strain of *Enterobacter cloacae*, a fermentative hydrogen-producing bacterium. Contrary to our results, the bioaugmented set-ups of both studies started to produce more methane or biogas after a few days, and the difference between bioaugmented and non-bioaugmented set-ups lasted until the end of the experiment. The reason why in our study, the methane production in the bioaugmented set-ups with pre-treated straw was higher right from the beginning could be that the alkaline pre-treated straw and the microorganisms from the enrichment cultures were pre-incubated for 20.5 h before they were mixed with the inoculum slurry.

The fact that in our study, the methane yields of bioaugmented and non-bioaugmented set-ups were similar at the end could either indicate that the added microorganisms could not use more of the lignocellulolytic substrate, but could use it faster than the microorganisms of the inoculum slurry or that the added microorganisms could not compete with the other microorganisms of the inoculum slurry under the environmental conditions for a longer period. The survival and competition of the added microorganisms is crucial for the success of a bioaugmentation procedure. This is of special importance when the added microorganisms were enriched from another environment than the one that they are added to, as in our case. The chances for the survival of these newly-introduced microorganisms are generally low, since they are often worse adapted to the abiotic and biotic conditions than the autochthonous microorganisms [21,41]. 

### 4.3. Effect of Bioaugmentation on Anaerobic Fermentation in a Leach-Bed Fermenter

Lignocellulosic substrates, like straw, can efficiently be digested in leach-bed reactors [42,43]. The positive effect of alkaline substrate pre-treatment on biogas production in leach-bed fermenters was already reported, aiming at methane production as the major target [44,45]. In this study, the investigations focused on acidogenic processes resulting mainly in the production of VFA. In the experiments P1–P3, a higher VFA production was observed compared to the reference experiment with untreated straw. 

Methane production was detected only in one experimental set-up (P2). On the one hand, this was the consequence of the acidogenic nature of inoculum StI in experiment P1. In P2 and P3, S37°C was used as the inoculum, which contained also methanogens [30]. Nevertheless, methanogenesis in the P2 experiment was observed unexpectedly, because of unfavorable conditions in terms of the low pH values. Methane production led probably to lower gas production in P2 compared to P3 due to hydrogen and carbon dioxide conversion to methane, which is connected to a gas volume reduction. Generally, due to the high TS content in the reactors, VFA production could have been preferred [46]. Methanogenesis initiation in leach-bed systems can be substantially affected by spatial heterogeneities in the feedstock and the formation of pH-neutral niches [47]. Karakashev *et al.* [48] described community compositions in industrial-scale reactors, where the inoculum community appeared to have no influence on the eventual one, at least regarding methanogens. This was similarly shown in this study, where the assertion of methanogens was observed only in one case, however, to a similar degree in both duplicates of P2. As a result, the methanogenesis initiation in P2 seemed to be not directly an effect of the microbial composition of the inoculum (S37°C). This hypothesis was supported by the different methanogenic community compositions observed in S37°C and P2 (Appendix, Figure A6). In one reactor, T-RF 57 predominated, which was tentatively assigned to the genus *Methanosarcina* based on *mcrA* sequence data from other lab-scale experiments (data not shown). T-RF 57 was not detected in the inoculum culture S37°C. However, rare community members not detected by T-RFLP analysis may become important when conditions turn favorable. Alternatively, this T-RF could have originated from the autochthonous community of pre-treated straw. *Methanosarcina* has been discussed as the “heavy duty methanogen” [49], and due to a high tolerance towards low-pH and high-VFA conditions, it seems to play an important role in initiation of methanogenesis under adverse conditions [50].

The inoculum can influence the anaerobic fermentation, e.g., Hu and Yu [51], using rumen fluid as the inoculum, observed an increased VFA production and no methane production from lignocellulosic feedstock. Furthermore, other researchers emphasized the role of inoculum selection on the AD of solid substrates [52,53,54,55]. In this study, two inocula from very different origins, *i.e.*, an alkaline environment in the case of S37°C and a biotechnological process at neutral and, later, acidic conditions in the case of StI, were applied. However, both experiments resulted in fermentation characteristics, which differed rather quantitatively than qualitatively, pointing at a stronger influence of the substrate than of the inoculum. In agreement with the results of the BMP assays, the effect of the inoculum choice on the fermentation was smaller than the effect of the alkaline pre-treatment procedure (Figure 3). Furthermore, the eventual bacterial community compositions of P1, P2 and P3 did not differ significantly, but at least, the bacterial communities of P2 and P3 were dissimilar to that of their inoculum S37°C (Appendix Figure A4). Accordingly, in our case, the eventual bacterial community compositions seemed to be rather independent of the inoculum type. This was surprising, as a pronounced metabolic effect of the inoculum choice was observed. An effect of the chemical composition of the inocula, at least regarding soluble matter, can be excluded, as both inocula were washed before addition. We can speculate that similar to the BMP assay, the initial activity of the inoculum communities may have influenced the hydrolysis and acidogenesis in the leach-bed systems substantially, while the eventual community composition after several days was more strongly shaped by the used substrate. The differences in the community compositions of untreated and pre-treated straw could thus be explained by the effect of the strong pre-treatment conditions, which only resistant microorganisms, like spore formers, could have survived. The alkali-tolerant culture S37°C could have been able to deal better with the alkaline conditions of the pre-treated substrate in the beginning, whereas with the ongoing fermentation process, the autochthonous microorganisms had the edge over the added microorganisms. 

## 5. Conclusions

In this study, the effect of the addition of alkali-tolerant, lignocellulolytic mixed cultures, enriched from soda lakes, on the anaerobic fermentation of Ca(OH)_2_-pre-treated wheat straw was determined with two different approaches: (i) with a BMP assay for biogas production; and (ii) with leach-bed reactors for VFA production. Additionally, the anaerobic fermentations of untreated and pre-treated wheat straw with standard inocula (without the alkali-tolerant cultures) were followed. Both approaches clearly showed that the pre-treatment with Ca(OH)_2_ alone improved the anaerobic degradation of the straw substantially. The additional positive effect by providing alkali-tolerant microorganisms to the systems was smaller, indicating that, at least in the studied systems, usual standard inocula can cope with the alkaline substrate. Furthermore, community analysis revealed that the alkali-tolerant microorganisms used for bioaugmentation did not survive in quantitatively relevant numbers in the leach-bed reactors. The BMP assays showed an improvement of the AD of pre-treated straw by the addition of alkali-tolerant microorganisms within the first week. Similarly, the fermentation of pre-treated straw for VFA production showed no lag-phase when an alkali-tolerant culture was used as inoculum compared to the standard inoculum having a lag-phase of two days. We therefore suppose that initially, the alkali-tolerant cultures could better cope with the alkaline substrate, whereas the microorganisms present in the inoculum slurry (BMP assay) or the autochthonous microorganisms from the straw itself (leach-bed reactors) prevailed in the long term. Nevertheless, the fermentation products in the leach-bed set-ups with bioaugmentation reached higher concentrations compared to the non-bioaugmented set-ups. Bioaugmentation with alkali-tolerant microorganisms may be convenient for shortening the start-up phase of anaerobic fermentation of alkaline pre-treated substrates. It remains to be investigated if these cultures can be beneficial in continuous systems, e.g., for bridging the adaptation time in case of substrate shifts.

## Appendix

For this experiment, the same AMPTS was used under the same conditions as described in the Experimental Section 2.3, with the following exceptions. As a substrate, 4.3 g of untreated straw (for the characteristics, see Table 2) were used, and 375.7 g of inoculum slurry (another load with the same composition as described in Section 2.3, pH value 7.5, TS = 3.3%_Fresh·Mass_, VS = 1.9%_Fresh·Mass_) were added. The microorganisms of S37°C and V37°C were suspended in 0.9% (w/v) NaCl solution, and 20 mL were added directly to each AMPTS reactor without any pre-incubation. Reactors without bioaugmentation received 20 mL 0.9% (w/v) NaCl solution only. As in the main experiment, the added cell number corresponded to approximately 1% of the total cell number of the inoculum slurry. The headspace of each AMPTS reactor was flushed with N_2_ for 30 s.

S37°C and V37°C were cultivated in 500-mL batches, as described in the Experimental Section 2.3, and were inoculated with the third transfer of S37°C and V37°C described by Porsch *et al.* [30]. The microorganisms were harvested as described in the Experimental Section 2.3, except that they were washed two times with 0.9% (w/v) NaCl solution.
